# The Effect of Vitamin E Supplementation in Postmenopausal Women—A Systematic Review

**DOI:** 10.3390/nu15010160

**Published:** 2022-12-29

**Authors:** Stepan Feduniw, Lidia Korczyńska, Konrad Górski, Magdalena Zgliczyńska, Monika Bączkowska, Maciej Byrczak, Jakub Kociuba, Mohamed Ali, Michał Ciebiera

**Affiliations:** 1 Department of Gynecology, University Hospital Zürich, 8091 Zürich, Switzerland; 2 Second Department of Obstetrics and Gynecology, Centre of Postgraduate Medical Education, 00189 Warsaw, Poland; 3 Department of Obstetrics, Perinatology and Neonatology, Centre of Postgraduate Medical Education, 01809 Warsaw, Poland; 4 Clinical Pharmacy Department, Faculty of Pharmacy, Ain Shams University, Cairo 11757, Egypt; 5 Department of Obstetrics and Gynecology, University of Chicago, Chicago, IL 60637, USA

**Keywords:** menopause, climacteric, vitamin E, tocopherol, hot flashes

## Abstract

Menopause is a physiological change in any woman. Nevertheless, its symptoms could be difficult to accept, and hormone therapy can be sometimes unattractive or contraindicated. Vitamin E components are phytoestrogens, so they are believed to be useful in some indications including menopause. This review aimed to assess the available evidence on the effectiveness of vitamin E in alleviating menopausal symptoms. The Pubmed/MEDLINE, Cochrane Library and Scopus databases were screened. All types of studies that assessed the effectiveness of vitamin E in alleviating menopausal symptoms were included. The PICO question was: “How does vitamin E supplementation affect menopausal symptom occurrence?” The PROSPERO ID number of this review is CRD42022328830. After quality assessment, 16 studies were included in the analysis. The studies were divided into three groups in which the influence of vitamin E on the genital syndrome of menopause, vasomotor symptoms and vascular and metabolic changes were assessed. Vitamin E influences postmenopausal hot flashes, vascular modulation, plasma lipid profile level and vaginal changes. Compared to vitamin E, estrogen administration leads to better clinical effects. Nevertheless, vitamin E might serve as additive to hormone therapy and its alternative in women with contraindications to estrogens. More quality data are necessary to draw final conclusions.

## 1. Introduction

Menopause is a condition diagnosed retrospectively, by determining the time of the last menstrual period after which the bleeding did not appear for the following 12 months [1]. The average age of menopause worldwide is about 51 years. However, menopause should not be considered as an exact time point. It should rather be perceived as the entire period of changes in the woman’s body functioning, resulting from the cessation of the ovarian hormones release. According to some experts, terms such as the menopausal transition or postmenopause reflect the meaning more precisely [2]. It is estimated that the number of postmenopausal women worldwide will be around 1.1 billion in 2025 [3]. Moreover, the increase in female life expectancy makes menopause an increasing part of a woman’s life. As menopausal symptoms may significantly reduce the quality of life (QoL), providing adequate care and treatment becomes a great clinical challenge worldwide [4].

Menopausal symptoms are related to hormonal changes in the woman’s body. The postmenopausal period is characterized by increased levels of follicle-stimulating hormone (FSH) and decreased levels of estrogen. The clinical picture is complex and affects the patients’ QoL at various levels. The symptoms may be divided into two main groups—vasomotor symptoms and genitourinary syndrome of menopause (GSM). Other symptoms include mood disorders, insomnia, memory or cognitive impairment and others [5,6].

The most common vasomotor symptoms include hot flashes and night sweats, often associated with sleep disorders. According to available data, hormone therapy is the most effective treatment in this indication [7,8]. Treatment should be tailored to the needs and expectations of each patient, considering the individual benefit-risk balance. Nonetheless, numerous patients refuse hormonal therapy due to the fear of its complications or other reasons [9,10,11]. Furthermore, non-pharmacological interventions, such as lifestyle modifications, showed some effectiveness and a significant placebo effect [12].

GSM is defined as a complex of symptoms associated with estrogen deficiency occurring during the menopausal transition. The clinical picture most commonly includes vaginal dryness, irritation, itching, urinary tract dysfunctions, and dyspaurenia. Despite its frequent occurrence, the syndrome is still underdiagnosed and often untreated. A significant problem is also attributed to either the women’s reluctance to seek consultancy or the unawareness of the disease where women are not perceiving the symptoms as a pathology and accepting them as an inexorable consequence of aging. This makes the diagnosis and the implementation of adequate treatment a great clinical challenge [13,14,15].

For many years the treatment of menopausal symptoms has remained the subject of interest. Menopause hormone therapy is contradicted in numerous cases, e.g., in patients with a history of hormone-dependent tumors, thromboembolism or selected heart diseases. The need for alternative therapeutic options is then substantial [16]. The personalization of therapy is another research direction which is extensively studied nowadays. Alternative drugs may also act as complementary to menopause hormone therapy [17]. Vitamin E is worth mentioning as an alternative/complimentary treatment. Some aut hors proposed the use of vitamin E, due to its antioxidant potential, as a treatment method in a group of patients who do not want or cannot have hormone therapy [18]. Vitamin E is a substance with a strong antioxidant effect. The group of vitamins E includes tocotrienols and tocopherols. Alpha-tocopherol is the most important in the human body, as it shows the greatest biological activity [19]. Vitamin E is fat soluble therefore is found in the human body in plasma lipoproteins. It is also a component of cell membranes. Vitamin E is an exogenous substance, with its dietary source being mainly vegetable oils, grains, meat, and eggs [20]. Due to the ubiquitous presence of vitamin E in food, its deficiency in the body is rare; it may be associated with the malabsorption of lipophilic vitamins. Vitamin E deficiency may be manifested as a tendency to hemolysis and, consequently, hemolytic anemia. Decreased immunity may also be a consequence of its deficiency. The immunosuppressive effect is explained by the improper functioning of immune cells, balance disturbance of the antioxidant system and so the unfavorable effect of free radicals. The anti-allergic, cardio- and neuroprotective or anti-cancer effects of vitamin E remain major subject of research [21,22]. Additionally, the biological effect of vitamin E is explained by the finding that tocopherols and tocotrienols, as phytoestrogens, are the modulators of estrogen receptors. Therefore, proposing the rationale of vitamin E potential utility in the treatment of menopausal symptoms [23].

There are also data concerning the possible effects of vaginal vitamin E local therapy in the alleviation of GSM symptoms and its potential as an alternative therapy to estrogen therapy. The results of several recent studies are promising as the benefits of short-term treatment and the effectiveness seem comparable to estrogen therapy. However, there is a need for further research to assess the long-term efficacy and safety [24]. There are also studies reporting the effectiveness of vitamin E in relieving vasomotor symptoms such as hot flashes [25,26]. However, data on the use of vitamin E in menopausal symptom treatment are largely incomplete and inconclusive. There are no clear guidelines regarding the treatment, its benefits, safety, or potential complications. Interestingly, according to the British National Institute for Health and Care Excellence (NICE) guidelines for the treatment of breast cancer published in 2022, the use of vitamin E is not recommended in the treatment of menopausal symptoms in patients with breast cancer [27].

Vitamin E is widely recommended by producers of supplements and ‘healthy food’ as a cure for everything. However, the results of studies on the benefits of its supplementation in disease prevention and safety assessment are inconclusive [28]. There are even reports of a possible harmful effect and an increase in the overall mortality in case of unjustified vitamin E supplementation [29]. The relationship between vitamin E and the carcinogenesis process was also a subject of research. Some of available studies found relationship between vitamin E supplementation and an increased risk of developing prostate cancer [30]. Still there is a concern that phytoestrogens, by acting on steroid receptors, may have an adverse effect on the risk of hormone-dependent cancers—especially breast cancer and endometrial cancer [31]. It is possible that the effect depends on the dose and duration of supplementation. However, several studies showed a protective effect on the development of breast cancer [32]. A relationship is also suspected between increased alpha-tocopherol levels and the occurrence of uterine fibroids [33].

The evidence for the efficacy and safety profile of phytoestrogens is limited, and the impact of its supplementation on health remains unclear. Therefore, the question concerning the effectiveness and use of vitamin E in the treatment of menopausal symptoms remains unanswered. The aim of this publication is to systematically review available literature on the effectiveness of vitamin E in the alleviation of menopausal symptoms.

## 2. Materials and Methods

### 2.1. Study Design

The current systematic review was performed according to the systematic reviews and meta-analyses (PRISMA) guidelines [34]. The systematic review protocol was registered in PROSPERO database with ID-number: CRD42022328830.

### 2.2. Search Strategy

The authors screened the following databases: Pubmed/MEDLINE, Cochrane Library and Scopus using the search strategy presented in Table 1.

### 2.3. Inclusion Criteria

All types of evaluative study designs were included and assessed. Two reviewers (SF and LK) independently screened the studies by the title, abstract and full text. Studies that met the selection criteria were included. Every included study was assessed to the group (0 = not relevant, 1 = possibly relevant, and 2 = very relevant). Only publications that scored at least 1 point were included in the study. Any disagreement was discussed and resolved by the third researcher (MC).

- Types of studies: Only original papers according to study designs were eligible for inclusion.
- Types of participants: Perimenopausal women.
- Types of exposure: Vitamin E intake or its level in the human blood serum.
- Types of outcome measures: Perimenopausal or postmenopausal symptoms would be assessed.

Editorials, newspaper articles, and other forms of popular media were excluded. Failure to meet any of the above eligibility criteria resulted in the exclusion from the review, and a third independent reviewer resolved any apparent discrepancies resulting from the selection process. Not appropriate study and control group assessment were exclusion criteria.

### 2.4. Data Extraction

**Figure 1:**
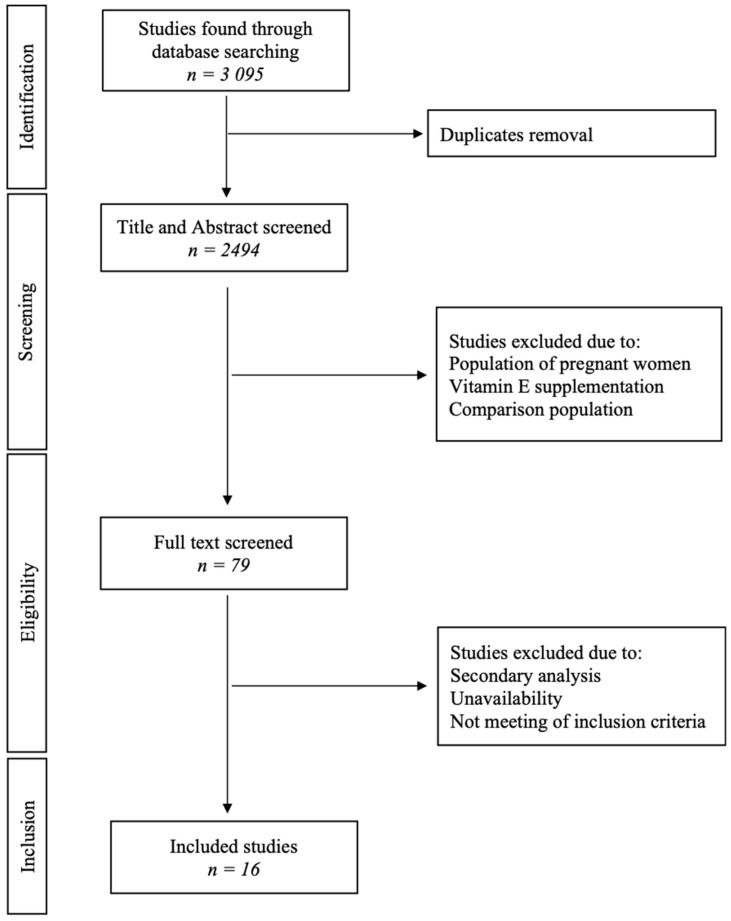
PRISMA systematic review flow diagram.

The PICO question was, “How does vitamin E supplementation affect menopausal symptom occurrence?” Population (P) Perimenopausal women with additional supplementation of vitamin E. Intervention (I) Additional Vitamin E supplementation. Comparison (C) Perimenopausal women without additional supplementation of vitamin E. The outcome (O) Perimenopausal symptoms. Studies (S) included in the analyses were retrospective or prospective. The PRISMA diagram was made according to Reporting Items for Systematic Reviews and Meta-Analyses: The PRISMA Statement and was shown in Figure 1 [35].

### 2.5. Quality Assessment and Risk of Bias

The risk of bias was assessed independently by two authors (SF and LK) using the Newcastle-Ottawa scale [36]. The third reviewer (MC) resolved apparent discrepancies in the selection process. In general, the studies included were of low to moderate quality. It was presented in Supplementary Materials Table S1.

### 2.6. Synthesis of Results

Due to the heterogeneity of the included studies, it was impossible to perform quantitative synthesis. Nevertheless, all prediction values of the AI methods of the included studies were compared in the groups and presented in Table 2, Table 3 and Table 4.

## 3. Results

After quality assessment, applying the inclusion and exclusion criteria, 16 studies were included in the final analysis [25,26,37,38,39,40,41,42,43,44,45,46,47,48,49,50].

To facilitate the analysis, the works were divided into three thematic groups: studies on the influence of vitamin E on vaginal atrophy, on vasomotor and general symptoms and on vascular changes and metabolic profile. Table 2, Table 3 and Table 4 present the main characteristics of the included studies. 

The first group included four studies assessing the influence of vitamin E on the occurrence of atrophic vaginitis (Table 2) [37,38,39,40].

The second group included four studies assessing the influence of vitamin E on vasomotor and other general, especially psychiatric and neurological symptoms in postmenopausal women. Symptoms also included in this group were hot flashes, nervousness, headaches, paraesthesia, insomnia, melancholia, vertigo, fatigue, arthralgia, myalgia, or palpitations (Table 3) [25,26,41,42]. To assess menopausal symptoms and their severety a lot of scales could be used. In included studies, Blatt-Kupperman menopausal index (with symptoms: nervousness, headaches, paraesthesia, insomnia, melancholia, vertigo, fatigue, arthralgia, myalgia, or palpitations) and Greene climacteric scale (assessing vasomotor, somatic, depressive and anxiety symptoms) were used [51,52]. 

The last group included nine studies assessing the influence of vitamin E on the vascular changes and the lipid profile (Table 4) [41,43,44,45,46,47,48,49,50]. Study of Cancelo Hidalgo et al. already included in the second group was also included in this group since it assessed both aspects. 

## 4. Discussion

Vitamin E influence on menopausal symptoms was first shown in 1995 [43]. Since then only few evidence was shown. The assessed 16 studies vary a lot regarding of symptoms described, the route of vitamin E application, its doses, and the method of evaluation of the results. 

### 4.1. Atrophic Vaginitis

It has been proven that the polyunsaturated cell membrane phospholipids are safeguarded by vitamin E. This effect was shown on the repair function of the human epithelium in the skin and other parts of the body [53]. Both vitamin E and estrogens are important in building of collagen, membrane functioning and metabolism of the cells [54,55]. Accordingly influence of vitamin E on menopausal atrophic changes of vaginal epithelium is very probable.

Four group of authors analysed the influence of vitamin E on atrophic vaginitis. In all trials the vitamin E was administered vaginally. 

In both studies, Ziagham et al. from 2012 and from 2013 used the same intervention [37,38]. In the publication from 2013 the superiority of preparations with vitamin E over placebo in terms of both alleviating the symptoms of atrophy and lowering the pH and improving the maturation index has been proven. The publication from 2012 shows the superiority of hyaluronic acid over vitamin E when it comes to alleviating symptoms and the maturation index, with no differences in the effect on vaginal pH between the preparations.

Research published by Parnan Emamverdikhan et al. and Golmakani et al. were performed on the same group of patients. They proved both objective (vaginal maturation value) and subjective (Abbreviated Sexual Function Questionnaire) improvement in vaginal atrophy caused by the use of vaginal formulation containing vitamin E. Nevertheless, conjugated estrogen cream was found to be superior to vitamin E formula in terms of amelioration of laboratory criteria.

### 4.2. Vasomotor, Neurologic and Psychiatric Symptoms of Menopause

Hot flashes, also known as vasomotor symptoms, are one of the most common menopausal symptoms which largely impair the QoL. They affect up to 60–80% of women with menopause and persist in about one-third of patients after reaching the age of 60 [56,57]. As the expected length of life in developed countries increases significantly and raising number of women stay active in their professional and personal life, an impairment of the QoL due to hot flashes is a real clinical problem. There are several modalities reducing the intensity and frequency of vasomotor symptoms. The use of hormone therapy is the most effective therapeutic option, but it might be associated with several risks and contraindications [58]. Therefore, this treatment cannot be offered to all menopausal women. The most problematic group of patients includes women after oncological treatment, especially those with a history of breast cancer. Due to chemotherapy or bilateral oophorectomy, they are often abruptly exposed to estrogen deficiency, which may lead to a higher incidence, frequency, and severity of hot flashes. Hormone therapy is contraindicated in patients with a history of estrogen-dependent cancers, e.g., breast cancer. Additionally, they often receive tamoxifen as an adjuvant agent. The reported incidence of hot flashes is especially high and reaches 80% in this particular group. Up to 30% of such patients declared severe symptoms [59]. Non-hormonal therapy, such as selective serotonin reuptake inhibitors (SSRIs) or serotonin norepinephrine reuptake inhibitors (SNRIs), might be a good option to consider in such women. Available data confirmed the reduction of the intensity and severity of hot flashes up to 70–80% with the use of these agents [56]. Conversely, the limitation of this type of therapy in tamoxifen-receiving patients is related to the fact that some SSRI/SNRI agents (especially paroxetine and fluoxetine) inhibit CYP2D6 which leads to the reduction of the level of active tamoxifen metabolite (endoxifen) [60,61]. Considering the above, vitamin E offers an opportunity of being an alternative modality. Some research was conducted to assess the efficacy of vitamin E in the reduction of hot flashes. A recent meta-analysis showed that vitamin E combined with omega 3 fatty acids significantly reduced the intensity of hot flashes compared to placebo [62]. According to the authors, the main limitation was a low number of RCTs and small sample size in the analyzed studies. As the data for the general population are encouraging, breast cancer survivors could also benefit from this type of therapy. Vitamin E did not cause side effects compared to gabapentin or clonidine for example, so it is worth investigations as a first-line treatment [63]. Attempts are also made to treat intense symptoms using acupuncture, soy, red clover or black cohosh, but there is currently no evidence to confirm their effectiveness. Good quality data are needed to establish the benefits of vitamin E in the reduction of both the intensity and severity of hot flashes in menopausal woman, especially in the group of patients for whom the type of therapy should be selected very carefully in the context of their medical history.

Four studies investigated the influence of vitamin E on the vasomotor symptoms of menopause, as well as neurological and psychiatric changes during menopause. Cancelo Hidalgo et al. proved a significant effect of an oral supplement containing both 20 and 10 mg/day vitamin E on the improvement of hot flushes and insomnia as well as other parameters measured in the Blatt-Kuperman scale, however given supplement also contained isoflavones and primrose oil [41]. Ziaei et al. and Ataei-Almanghadim et al. supplemented 400 IU/day to included patients. In these studies the beneficial effect on hot flushes was also confirmed [25,26]. On the other hand, Farshbaf-Khalili et al. using the Greene scale, proved the beneficial effect of vitamin E supplementation on anxiety and the overall score on this scale by supplementation of 500 mg of vitamin E twice a day [42]. In all asesed studies a positive effect on discussed menopausal symptoms was shown. Nevertheless, the doses used differed. The release of the symptoms was shown in all studies. As effect of vitamin E might be doses-dependend, the toxical influence on the organism should not be forgotten. Further studies should be performed to estimate the most effective and the less harmful dose of vitamin E in vasomotor, neurologic or psychiatric symptoms release.

### 4.3. Influence on the Lipid Profile and Vascular Changes

It is well known that the incidence of cardiovascular disease (CVD) increases dramatically after menopause [64]. While it is related to the natural aging of the body, but hormonal changes play an invaluable clear role. Increase in cardiovascular risk was found to be particularly pronounced in women with premature ovarian failure [65]. Lipid metabolism and its disorders are among the most important modifiable risk factors for CVD. Menopause is a period in women’s lives which is associated with a significant deterioration of the lipid profile. A recent meta-analysis conducted by Ambikairajah et al. revealed that postmenopausal women had higher levels of triglycerides (TG) and low-density lipoproteins (LDL) compared to premenopausal women [66]. Total cholesterol, LDL and TG concentrations were lower in premenopausal women than in men, while high density lipoprotein cholesterol (HDL) concentration was higher than in men [67,68]. Thereafter, the LDL fraction in women was increased by about 2 mg/dL per year between the ages 40 and 60, which included both premenopausal age and mainly postmenopausal years [69,70]. Studies on changes in HDL concentration are inconsistent [67,71]. The above-described changes are mainly due to both estrogen deficiency and the increase in levels of free androgens [65]. The effects of menopause on the lipid profile may be partially reduced by hormone replacement therapy, mainly in terms of reducing LDL levels and increasing HDL levels [72,73].

Vitamin E is considered an element in dyslipidaemia treatment regimen. It decreased lipid peroxidation both in vitro and in vivo by breaking chain propagation [72]. Moreover, it was demonstrated that vitamin E suppressed atherogenesis via influencing the endothelial and arterial smooth muscle cells [73,74]. In addition, supplementation with vitamin E ameliorated LDL resistance to oxidation propagation [74]. Although research results are inconsistent, some studies indicated that vitamin E supplementation might improve selected parameters of the lipid profile [46,50,75]. Few large-scale studies of dietary vitamin E intake showed a positive effect in reducing the risk of coronary heart disease in both men and women [76,77]. It was also confirmed in postmenopausal women with only dietary intake [78]. Wang et al. studied a group of Chinese women with metabolic syndrome and demonstrated that four-month of vitamin E supplementation at a dose of 300 IU/day lowered total cholesterol (TC), although it should be mentioned that it also significantly reduced HDL [79]. Vitamin E and omega-3 fatty acids co-supplementation was proved to reduce the levels of VLDL [74]. Moreover, several studies showed a beneficial effect on lowering the risk of cardiovascular disease, especially in population at risk with pre-existing diseases [80,81]. Conversely, it should be mentioned that apart from articles that showed no significant effect of the intake of vitamin E on the lipid profile and cardiovascular risk, there are also studies that indicated a negative effect of vitamin E supplementation in some groups of patients. A study conducted in a male smokers group showed that alpha-tocopherol supplementation was associated with more deaths from hemorrhagic stroke compared to placebo [82]. Furthermore, a meta-analysis of clinical trials showed that the majority of trials testing high-dose supplementation of vitamin E demonstrated an increase in all-cause mortality when compared to the control group [29].

Nine of the included studies investigated the influence of vitamin E on the lipid profile level and other vascular changes. Studies from Rezasoltani et al., Cancelo Hidalgo et al. and Rasool et al. did not show the influence vitamin E supplementation on lipid profile and/or postmenopausal arterial remodulation, respectively [47,50]. On the other hand, Koh et al. also demonstrated no effect of vitamin E on lipid profile and vascular disease markers, however showed improvement in arterial endothelium-dependent vasodilator responsiveness [46]. Ushiroyama et al. proved the superior effect of Wen-jing-tang over 600 mg/day vitamin E intake on the regulation of peripheral blood flow [48]. Alves Luzia et al. and Wander et al. have proven the positive influence of combined treatment with vitamin E with fish oil on lipid profile and oxidation, whereas Cancelo Hidaldo et al. showed improvement in vasomotor symptoms after vitamin E, isoflavones and primrose oil supplementation [41,44,49]. Inal et al. proved that combined administration of estrogens and vitamin E improve lipid profile, whereas Guetta et al. found independent positive effect of these substances on lipid oxidation [43,45].

### 4.4. Summary of Evidence

Vitamin E was shown by some studies to influence postmenopausal vasomotor symptoms, plasma lipid profile, vascular, psychiatric, neurological as well as and vaginal changes. In competetive studies the superiority of estrogens vs. vitamin E in menopausal symptom reduction was apparent. Nevertheless, vitamin E seem to be an excellent addition to hormone therapy or effective as monotherapy in women contraindicated to estrogen therapy. Estrogen/vitamin E combination treatment could lead to a better effect of the hormone therapy or to fewer side effects since the used dose of estrogens should be reduced. Very few studies were conducted to evaluate the combined therapy (estrogens with vitamin E) [40,46]. Moreover, no studies compared the combined therapy of hyaluronic acid with vitamin E, which could be a better option than hyaluronic acid alone in patients contraindicated to estrogens. Conversely, vitamin E’s effectiveness is difficult to conclude from this review because of the heterogeneity of the doses. The mean vitamin E dose was difficult to assess as different routesof medicament application, different compounds and different units (IU or mg) were used.. Only one study was identified to compare the effectiveness of different vitamin E doses, so future investigation in this field could offer the medical provider detailed information about vitamin E effectiveness and safety.

Moreover, antidepressant medications, including SSRI and SNRI, are used to decrease psychosomatic and vasomotor symptoms [83]. As was shown in the review, vitamin E alone could influence psychological issues [25,26,41]. The additional influence of the placebo effect improves the women’s well-being. A combination of these three methods could lead to a decrease in the psychological and vasomotor symptoms of menopause.

### 4.5. Limitations

Our study has several limitations. First of all, there was a problem with the synthesis of the results because of the heterogeneity of the studies. Therefore, several deviations in the groups were made to approximate the similar outcomes of the assessed studies. Unfortunately, this was insufficient because of methodological differences and incomparable doses of vitamin E and heterogeneity of control groups, where sometimes estrogens were used. Moreover, the average quality of included studies was low to moderate, with very small groups, as only five studies have participant rate over 50 persons in each control and study group. To better interpretation of influence of vitamin E on menopause symptoms assessment in three groups was performed.

## 5. Conclusions

Vitamin E influences postmenopausal symptoms like hot flashes, vascular modulation, plasma lipid profile level and vaginal changes. In comparison with vitamin E, estrogen administration leads to better clinical effects. Nevertheless, vitamin E might become an option as an addition to standard hormone therapy. It might be used as an alternative compound in symptomatic patients with contraindications to estrogens (e.g., cancer survivors). More good quality data are necessary to draw final conclusions.

## Figures and Tables

**Table 1:** Search strategy.

Databse	Search Strategy
PubMed/MEDLINE	(“Vitamin E”[Mesh] OR (vitamin* AND e) OR tocopherol* OR tocotrienol*) AND (“Menopause”[Mesh] OR menopaus* OR postmenopaus* OR perimenopaus* OR premenopaus* OR climacter*)
Scopus	TITLE-ABS-KEY ((vitamin* AND e) OR tocopherol* OR tocotrienol*) AND TITLE-ABS-KEY (menopaus* OR postmenopaus* OR perimenopaus* OR premenopaus* OR climacter*)
Cochrane Library	#1”Vitamin E”[Mesh] #2 (vitamin* AND e) OR tocopherol* OR tocotrienol* #3 “Menopause”[Mesh] #4 menopaus* OR postmenopaus* OR perimenopaus* OR premenopaus* OR climacter* AND (#1 OR #2) AND (#3 OR #4) + Trials

All searches were conducted on 1 August 2022 with languages restricted to English, German or Polish and no publication time limits were imposed.

**Table 2:** Characteristics of included studies concerning atrophic vaginitis.

Study	Character of the Study	Number of Participants		Outcomes
		Study Group	Control Group	
Ziagham et al. (2012) [37]	The double-blinded randomized controlled trial	20 postmenopausal women received vaginal tablets containing 1 mg vitamin E for 8 weeks—daily for 2 weeks, and subsequently, one suppository three days a week	20 postmenopausal women received vaginal tablets containing 5 mg of hyaluronic acid sodium salt for 8 weeks—daily for 2 weeks, and subsequently, one suppository three days a week	Both interventions provided relief of vaginal symptoms, however vaginal hyaluronic acid group was more effective in reducing the genitourinary symptoms of menopause than vitamin E.
Ziagham et al. (2013) [38]	The double-blinded randomized controlled trial	20 postmenopausal women received vaginal suppositories containing 1 mg of vitamin E for 8 weeks—daily for 2 weeks, and subsequently, one every other day	22 postmenopausal women received placebo suppositories with semisynthetic fatty acid triglyceride (placebo) for 8 weeks—daily for 2 weeks, and subsequently, one every other day	Vaginal vitamin E is more effective in reducing the genitourinary symptoms of menopause than placebo.
Parnan Emamverdikhan et al. (2016) [39]	The interventional, clinical trial	26 postmenopausal women received 100 IU vitamin E vaginal suppositories for 12 weeks—daily for 2 weeks and subsequently twice a week	26 postmenopausal women received 0.625 mgconjugated estrogen in vaginal creamfor 12 weeks daily for 2 weeks and subsequently twice a week	Estrogens are more effective in atrophic vaginitis. Nevertheless, vitamin E improve the laboratory signs of vaginal atrophy.
Golmakani et al. (2018) [40]	The single-blinded randomized controlled trial	26 postmenopausal women received 100 IU vitamin E vaginal suppository for 12 weeks—daily for 2 weeks and subsequently twice a week	26 postmenopausal women received 0.625 mg conjugated estrogen in vaginal cream for 12 weeks—daily for 2 weeks and subsequently twice a week	Vitamin E and estrogens have similar improving potential of sexual function of postmenopausal women.

**Table 3:** Characteristics of included studies concerning vasomotor, neurological and psychiatric symptoms of menopause.

**Study**	**Menopause** **Symptoms**	**Character of the Study**	**Number of Participants**		**Outcomes**
			**Study Group**	**Control Group**	
Cancelo Hidalgo et al. (2006) [41]	Menopausal symptoms (Blatt-Kupperman: menopausal index)	The interventional clinical trial	478 postmenopausal women received isoflavones 120 mg, primrose oil 880 mg and vitamin E 20 mg/day for 6 months.	447 postmenopausal women received isoflavones 60 mg, primrose oil 440 mg g and vitamin E 10 mg/day or 6 months.	Both doses of supplementation of isoflavones, primrose oil and vitamin E had positive influence on decreasing the menopausal symptoms like nervousness, hot flushes or insomnia. (The influences on methabolic changes was observed and will be described in Table 4).
Ziaei et al. (2007) [25]	Hot flashes	The placebo double blind-controlled trial	51 postmenopausal women received placebo capsule for 4 weeks followed by vitamin E softgel capsule (400 IU/day) for 4 weeks		The reduction in the intensity and frequency of hot flushes is significant greater after vitamin E supplementation than during placebo intake.
Ataei-Almanghadim et al. (2019) [26]	Menopausal symptoms (hot flashes, anxiety, sexual function)	The triple blind randomised controlled trial	29 postmenopausal women received capsules with vitamin E (400 IU/day) for 8 weeks.	30 postmenopausal women received 1 g/day curcumin and 30 women received placebo for 8 weeks.	Vitamin E significantly decreased hot flashes in comparison to placebo. The first significant effect was noticed after 8 weeks therapy. No impact on anxiety, sexual function, or menopausal symptoms was found.
Farshbaf-Khalili et al. (2022) [42]	Symptoms of menopause (Greene climacteric scale): anxiety, and sexual dysfunction	The triple blind randomised controlled trial	27 postmenopausal women received 500 mg capsule of vitamin E twice a day for 8 weeks.	26 postmenopausal women received 1 g/day curcumin and 28 women received placebo (1 g/day microcrystalline cellulose) for 8 weeks.	Vitamin E reduces general menopause symptoms and anxiety in comparison to placebo and curcumin.

**Table 4:** Characteristics of included studies concerning lipid profile and vascular changes.

Study	Menopause Symptoms	Character of the Study	Number of Participants			Outcomes
			Study Group	Control Group		
Guetta et al. (1995) [43]	Plasma lipid profile levels (LDL-C, HDL, TC, and TG)	The randomised, interventional clinical trial	10 postmenopausal women received vitamin E monotherapy (800 IU/day) for 6 weeks	9 postmenopausal women received a 17 beta-estradiol (0.1 mg/day) patch monotherapy (changed every 3 days) for 3 weeks	During combined phase all included subjects (n = 19) took vitamin E 800 IU/day for 6 weeks, and used the 17B-estradiol patch (changed every 3 days) for the last 3 weeks of this period	Combined administration of estradiol and vitamin E decreases LDL oxidation with no synergism.
Wander et al. (1996) [44]	Copper-catalysed oxidation of LDL	The double-blind crossover trial	48 postmenopausal women received vitamin E (0, 100, 200, and 400 mg/day of a-tocopherol acetate) in different time for 4 weeks, as by the end of the study each subject had received all four doses of vitamin E. 24 women received no estrogens.	48 postmenopausal women received placebo for 4 weeks. 22 women used oral therapy (0.625 mg estrogens and 10 mg medroxyprogesterone), one used a transdermal patch, and one received estrogen injections.		Vitamin E provides protection LDL from copper-catalysed oxidation. The usage of estrogens and fish oil independently decreases LDL modification. This process is dependent on the vitamin E doses.
Inal et al. (1997) [45]	TC, HDL, VLDL, LDL, MDA, SOD and GSH-Px levels.	The randomised, interventional clinical trial	22 postmenopausal women received received transdermal estradiol (3 weeks a month) and medroxyprogesterone acetate (10 mg/day) (during the last 10 days of treatment) and vitamin E (600 mg/day) for 6 months. Levels of blood lipids was compared to premenopausal women.	22 postmenopausal women received transdermal estradiol (0.05 g/day) for 6 months (3 weeks a month). 22 postmenopausal women received transdermal estradiol (3 weeks a month) and medroxyprogesterone acetate (10 mg/day) (during the last 10 days of treatment). The duration of the study was 6 months. Levels of blood lipids was compared to premenopausal women.		Combined therapy with estradiol, medroxyprogesterone acetateand vitamin E leads to improvement in lipid profile.
Koh et al. (1999) [46]	Plasma lipid profile levels (LDL-C, HDL, TC, and TG)	The double-blind, 3-period crossover study	28 postmenopausal women received vitamin E (800 IU/day) or a combination of the both therapies per day for each of three 6-week treatment periods, with 6 weeks off all therapies between treatment periods.	28 postmenopausal women received conjugated equine estrogens 0.625 mg/d and placebo or a combination of the both therapies per day for each of three 6-week treatment periods, with 6 weeks off all therapies between treatment periods.		Vitamin E as a supplement to estrogen therapy improves arterial endothelium-dependent vasodilator responsiveness consistent with increased nitric oxide.
Rasool et al. (2003) [47]	arterial stiffness, blood pressure	The randomized, crossover, double-blind, placebo-controlled clinical trial	10 postmenopausal women received vitamin E (400 IU/day) for 10 weeks.	10 postmenopausal women received a placebo for 10 weeks.		Vitamin E does not affect arterial stiffness and blood pressure in postmenopausal women.
Ushiroyama et al. (2006) [48]	Chilly sensation. Blood flow measured by laser Doppler under the jaw, in the middle finger, and in the third toe.	The randomised, interventional clinical trial	60 postmenopausal women received vitamin E (600 mg tocopherol nictinate/day) for 8 weeks.	60 postmenopausal women received a Wen-jing-tang (7.5 g/day) for 8 weeks.		Wen-jing-tang more effectively improves, in comparison to vitamin E, blood flow in peripheral tissue and is more effective in treatment of chilli sensations.
Cancelo Hidalgo et al. (2006) [41]	Metabolic changes (weight, blood pressure, triglycerides and LDL-level), vasomotor symptoms	The interventional clinical trial	478 postmenopausal women received isoflavones 120 mg/day, primrose oil 880 mg/day and vitamin E 20 mg/day for 6 months.	447 postmenopausal women received isoflavones 60 mg/day, primrose oil 440 mg/day g and vitamin E 10 mg/day for 6 months.		Vitamin E, isoflavones and primrose supplementation have no influence on weight or blood pressure. Level of triglycerides and LDL-cholesterol levels tends to decrease, however not significantly
Alves Luzia et al. (2015) [49]	Plasma lipid profile levels (LDL-C, HDL, TC, and TG)	The randomised placebo-controlled trial	19 postmenopausal women received vitamin E (400 IU/day) and fish oil for 3 months.	18 postmenopausal women received a placebo for 3 months. 22 postmenopausal women received fish oil for 3 months.		Supplementation of Fish oil plus vitamin E decreases TC and LDL blood level.
Rezasoltani et al. (2021) [50]	Plasma lipid profile levels (LDL-C, HDL, TC, and TG)	The double-blind, placebo-controlled, randomized, cross-over. Phase I/II trial	41 postmenopausal women received vitamin E (400 IU/day) for 4 weeks and after an 8-day pause period, placebo for next 4 weeks.	42 postmenopausal women received a placebo for 4 weeks and after an 8-day pause period, vitamin E for next 4 weeks.		Vitamin E showed no significant influence on the lipid profile in menopausal women.

TC—Total cholesterol, TG—triglycerides, HDL—high-density lipoprotein cholesterol, VLDL—very low-density lipoprotein cholesterol, LDL—low-density lipoprotein cholesterol, LDL-C—low-density lipoprotein cholesterol, MDA—malondialdehyde, SOD—superoxide dismutase, GSH-Px—glutathione peroxidase, IU—International Units.

## Data Availability

Not applicable.

## Supplementary Materials

The following supporting information can be downloaded at: https://www.mdpi.com/article/10.3390/nu15010160/s1. Table S1. The Newcastle-Ottawa scale for quality assessment of included studies.
