# Supplementary material for: The Effect of Vitamin E Supplementation in Postmenopausal Women—A Systematic Review
Source: Nutrients. 2022 Dec 29;15(1):160. doi: 10.3390/nu15010160 (PMC9824658; doi:10.3390/nu15010160)
Supplement: Supplementary file 1 [file nutrients-15-00160-s001.zip › nutrients-2059102-supplementary.pdf]

Supplementary Table S1. The Newcastle-Ottawa scale for quality assessment of included studies

Item	Ziagham et al. (2012)	Ziagham et al. (2013)	Parnan Emamverdikhan et al. (2016)	Golmakani et al. (2018)	Cancelo Hidalgo et al. (2006)	Ziaei et al. (2007)	Ataei-Almanghadim et al. (2019)	Farshbaf-Khalili et al. (2022)	Guetta et al. (1995)	Wander et al. (1996)	Inal et al. (1997)	Koh et al. (1999)	Rasool et al. (2003)	Ushiroyama et al. (2006)	Alves Luzia et al. (2015)	Rezasoltani et al. (2021)
A **Selection**																
Exposed truly representative of avarage	0	0	0	0	1	0	1	0	0	1	0	0	0	1	0	1
Selection of non-exposed from the same community	0	1	0	0	0	0	1	1	0	0	0	0	1	0	1	1
Exposure of ascertained by secure record or interview	1	1	1	1	1	0	1	1	1	1	1	1	1	1	1	1
Demonstration of outcome of interest not present at the start of the study	1	1	1	1	1	1	1	1	1	1	1	1	1	1	1	1
B **Comparability**																
Study controls for other variables	1	1	1	1	1	0	1	1	1	0	1	0	1	1	1	1
C **Outcome**																
Follow up long enough for outcome to occur	1	1	1	1	1	0	1	1	0	0	1	1	1	1	1	0
Complete follow up of all subjects accounted for	1	1	1	1	0	1	1	1	1	0	1	0	1	1	1	0
Subject to follow up unlikely to introduce biases?	0	0	0	0	0	0	0	0	0	0	0	0	0	0	0	0
Asssessment of outcomes	1	1	1	1	1	1	1	1	1	1	1	1	1	1	1	1
**Score**	6	7	6	6	6	3	8	7	5	4	6	4	7	7	7	6
